# Layer-by-Layer Freezing of Nanoconfined Water

**DOI:** 10.1038/s41598-020-62137-1

**Published:** 2020-03-24

**Authors:** Yiqing Xia, Hyeyoung Cho, Milind Deo, Subhash H. Risbud, Michael H. Bartl, Sabyasachi Sen

**Affiliations:** 1 Department of Materials Science & Engineering, University of California at Davis, Davis, CA 95616 USA; 2 Department of Chemical Engineering, University of Utah, Salt Lake City, UT 84112 USA; 3 Department of Chemistry, University of Utah, Salt Lake City, UT 84112 USA

**Keywords:** Nanoscience and technology, Chemical physics

## Abstract

Nanoconfined water plays a pivotal role in a vast number of fields ranging from biological and materials sciences to catalysis, nanofluidics and geochemistry. Here, we report the freezing and melting behavior of water (D_2_O) nanoconfined in architected silica-based matrices including Vycor glass and mesoporous silica SBA-15 and SBA-16 with pore diameters ranging between 4–15 nm, which are investigated using differential scanning calorimetry and ^2^H nuclear magnetic resonance spectroscopy. The results provide compelling evidence that the extreme dynamical heterogeneity of water molecules is preserved over distances as small as a few angstroms. Solidification progresses in a layer-by-layer fashion with a coexistence of liquid-like and solid-like dynamical fraction at all temperatures during the transition process. The previously reported fragile-to-strong dynamic transition in nanoconfined water is argued to be a direct consequence of the layer-by-layer solidification.

## Introduction

Numerous research findings over the last three decades have shown that water confined in nanometer-sized pores, created in nanoarchitected solids, displays noticeably different behavior compared to bulk water^1–10^. Most interestingly, however, water unlike any other liquid, displays pronounced dynamical heterogeneity under nanoconfinement and freezes in a stepwise fashion. Furthermore, temperature-resolved measurements of the dynamics of nanoconfined water have suggested a fragile-to-strong liquid-liquid polyamorphic transition en route to freezing^11–13^. This nonconformist behavior of water has aroused intense scientific interest in probing the perplexing nature of the freezing dynamics of water in well-defined nanopores contained inside solid materials.

One of the most interesting and well-known effects of nanoconfinement is the depression of the freezing/melting point of water, which has been extensively studied by calorimetry^14–19^. The freezing point of nanoconfined water, for pore diameters larger than 2.5 nm, is found to decrease linearly with the increase in the inverse of an effective pore size following the Gibbs-Thomson (G-T) relation^20^. The G-T relation for the freezing point depression Δ*T*_*m*_ can be expressed as: $\Delta {T}_{m}=2{V}_{mb}{T}_{mb}{\gamma }_{sl}/\Delta {H}_{f}(R-t)$, where *V*_*mb*_, *T*_*mb*_and *γ*_*sl*_ are the molar volume, melting point and enthalpy of fusion of the bulk crystal, respectively, *γ*_*sl*_ is the solid-liquid interfacial energy, *R* is the pore radius and *t* is the thickness of the interfacial water layer.

Although the effects of pore shape, pore filling and pore wall on the water freezing/melting behavior have been studied extensively in the past^14,21,22^, the nature of the dynamics of nanoconfined water during the solidification process remains quite controversial^10–13,23–27^. Nevertheless, the majority of experimental and simulation studies indicate the presence of three spatial regimes for water confined in the pores of silica with pore diameters ranging between 2–20 nm, characterized by quite different structural and dynamical behavior^10^. The first regime is a ~0.3 nm thick statistical monolayer of water molecules that are bound to the pore surface and perform restricted rotational motion. This layer is believed to remain unfrozen even down to ~190 K. Beyond this layer, water molecules still remain strongly structured by the pore walls of silica up to a distance of ~1 nm from the wall. The mobility of this “shell” of water is significantly lower, while its density is higher, compared to that of bulk water. Experimental and simulation studies indicate that this shell of water displays density layering. On the other hand, the density and mobility of the water molecules in the “core” region beyond ~1 nm from the pore wall is believed to be similar to that of bulk water. Therefore, it is likely that upon cooling the core region will freeze first, followed by the shell region and thus, the latter will display a stronger effect of spatial confinement compared to the former. It is quite likely that instead of an abrupt change in the melting point across the core-shell boundary region, the effect of nanoconfinement will result in a gradual increase in melting point from near the pore wall towards the center. This hypothesis would be consistent with the observation of a long low-temperature tail in the melting endotherm in the differential scanning calorimetric (DSC) scans of water confined in porous silica^20^.

On the other hand, ^2^H nuclear magnetic resonance (NMR) spectroscopic studies of D_2_O confined in 2.0 nm diameter pores in mesoporous silica MCM-41 have shown the presence of heterogeneous dynamics with a bimodal behavior characterized by the coexistence of a relatively immobile and a highly mobile fraction of molecules below the DSC freezing point^28^. The relative fraction of the mobile molecules decreases with lowering of temperature, until nearly all molecules become immobile at temperatures below ~190 K. This coexistence of mobile and immobile molecules and the progressive increase in the immobile fraction with temperature also appear to be consistent with the abovementioned hypothesis of the gradual variation of the melting point across the core-shell boundary region. Here we report the results of a systematic study of the freezing dynamics of water confined in the nanopores of mesoporous silica and Vycor^TM^ with a wide range of pore diameter (~4–15 nm) and geometry (*see Methods section*) that is carried out using a combination of DSC and ^2^H NMR to test this hypothesis. We show that the gradual “layer-by-layer” freezing model is consistent with the kinetics of the solidification process observed in experiments and can also explain the recently reported observation of an apparent fragile-to-strong dynamic crossover in nanoconfined water.

## Melting point depression of nanoconfined D_2_O

**Figure 1:**
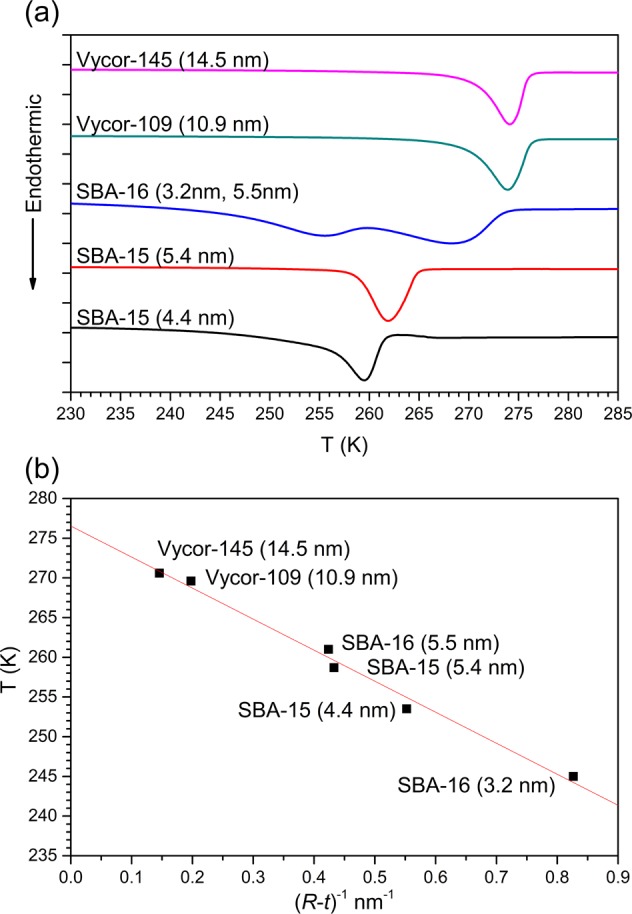
(**a**) DSC melting scans at 5 K/minute for D_2_O confined in Vycor-145, Vycor-109, SBA-15 and SBA-16. Corresponding pore diameters are given in the legend. (**b**) Melting point onsets determined from the DSC scans in (**a**) as a function of the inverse of the effective pore radius. Red straight line through the data points is a linear least-squares fit of the simplified Gibbs-Thomson equation (see text for details).

DSC heating curves for D_2_O in two mesoporous silica SBA-15 with regular cylindrical 4.4 and 5.4 nm diameter pores, in mesoporous silica SBA-16 with large spherical pores (5.5 nm diameter) connected by narrow cylindrical channels (3.2 nm diameter) and in two Vycor glass samples with tortuous channels with average diameters of 10.9 nm and 14.5 nm (denoted, respectively, as Vycor-109 and Vycor-145) are shown in Fig. 1a. While only one melting peak exists in the DSC scans of all samples with a single nanopore/channel size, two endothermic peaks are observed for the partially hydrated SBA-16 sample, corresponding to water confined in this bimodal pore structure. This is the first report, to the best of our knowledge, of the observation of two distinct freezing points for water nanoconfined in interconnected pores of different size. Considering the connectivity between the spherical and cylindrical pores in SBA-16, the observation of dual melting points in this system implies that under nanoconfinement strong dynamical heterogeneity of water molecules can be preserved even over such short distances of a few Å. The melting temperature *T*_*m*_ of confined D_2_O in these systems, corresponding to the onset of the DSC endothermic peak, strictly obeys the G-T relation and varies linearly with the inverse of the effective pore size (*R − t*) as shown in Fig. 1b. The least-squares fitting of melting points according to the simplified form of the G-T relation: $\Delta {T}_{m}=K/(R-t)$ yields fitting parameters $K=39.1\pm 2.1$ K ∙ nm and $\,t=0.39\pm 0.06$ nm, the latter being consistent with the thickness of the statistical monolayer of water molecules attached to the pore wall. Furthermore, this value of *t* for confined D_2_O is similar to that obtained for confined H_2_O^14^, where $t=0.38\pm 0.06$ nm, suggesting a similar interfacial bonding scenario for H_2_O and D_2_O. The experimental value of *K* is somewhat smaller than the theoretical constant ${K}_{th}=\frac{2{V}_{mb}{T}_{mb}{\gamma }_{sl}}{\Delta {H}_{f}}=50.4$ K ∙ nm, as obtained from using the values for bulk D_2_O, i.e. the melting temperature ${T}_{mb}=277.0$ K, specific volume ${V}_{mb}=0.9009\times {10}^{-6}$ m^3^/g, solid-liquid interfacial energy ${\gamma }_{sl}=31.7\times {10}^{-3}$ J/m^2^ and enthalpy of fusion $\Delta {H}_{f}=313.6$ J/g^29^. The deviation between the experimental and theoretical values of *K* is probably due to the sharp-interface approximation that is implicit in the G-T model. Finally, it is important to note that in all cases the DSC heating curve endotherms display a long low-temperature tail associated with the main peak, which is consistent with the scenario of gradual melting and progression of the solid-liquid interface from the pore wall towards the center, across the core-shell boundary.

## Freezing kinetics of nanoconfined D_2_O from ^2^H wideline NMR spectroscopy

**Figure 2:**
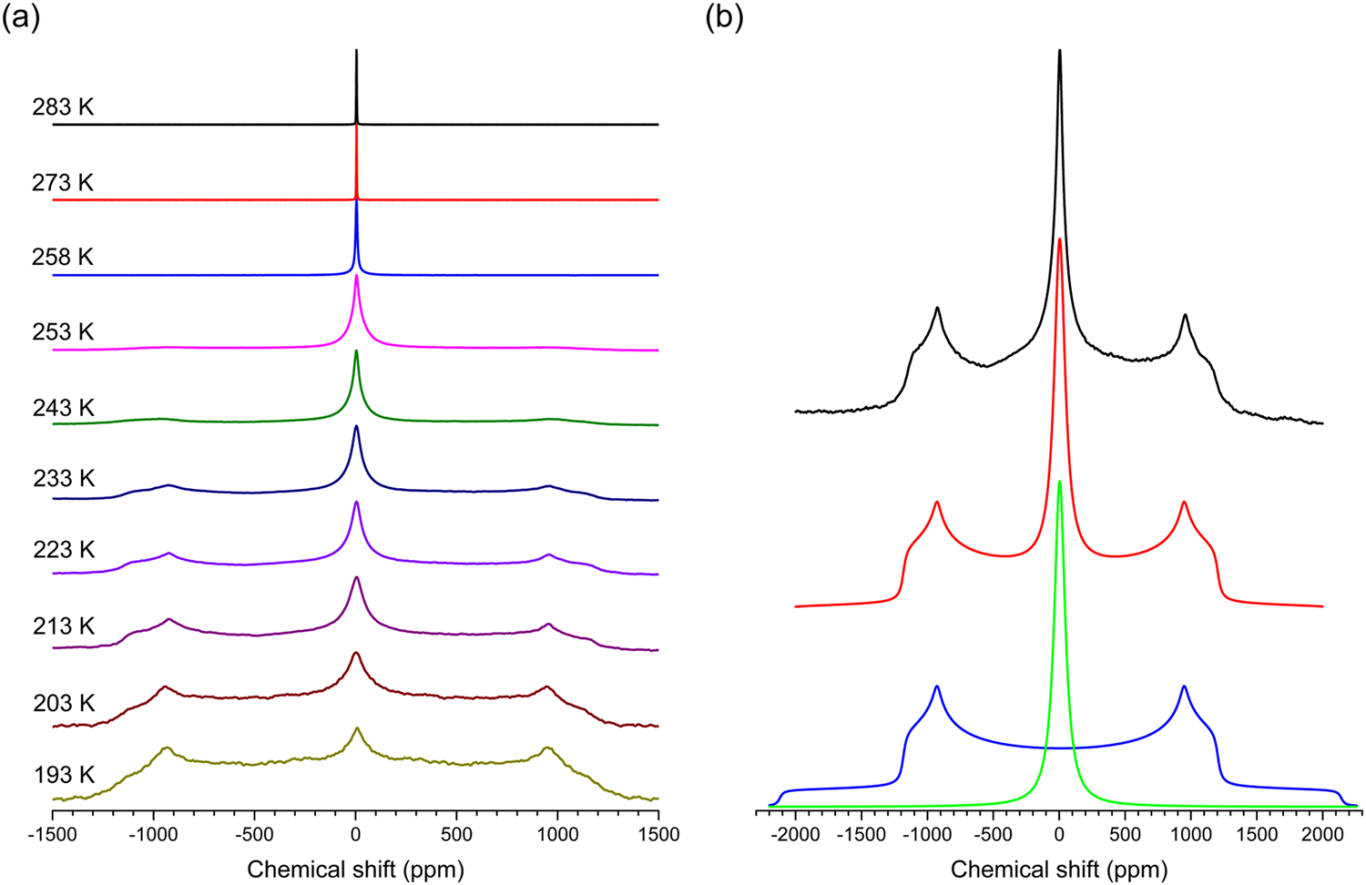
(**a**) Representative variable-temperature ^2^H NMR wideline spectra of D_2_O confined in Vycor-109. (**b**) Comparison between experimental (black line, top) and simulated (red line, middle) line shapes for the ^2^H NMR spectrum collected at 223 K from (**a**). Individual simulation components are shown in green and blue (bottom). The Lorentzian line (green) and the Pake pattern (blue) correspond to the liquid-like and solid-like fraction, respectively.

^2^H wideline NMR spectra were acquired for all samples of nanoconfined D_2_O over a temperature range of 193–283 K and representative spectra are shown in Fig. 2a for the Vycor-109 sample. Spectra collected during cooling and heating were found to be identical, indicating the absence of any hysteresis. At the highest temperatures the ^2^H NMR spectra are characterized by a narrow Lorentzian line indicative of rapid isotropic reorientation of D_2_O molecules, characteristic of liquid water. An additional broad powder-like pattern (Pake pattern) appears at temperatures below 263 K, which corresponds to a sub-population of molecules that are either completely rigid or performing rotational motion at a frequency on the order of a few kHz or less. Simulation of this Pake pattern yields a quadrupolar coupling constant C_Q_ of 215 ± 10 kHz and an asymmetry parameter η = 0.1 ± 0.02 at all temperatures for all samples (Fig. 2a). These values are in good agreement with the literature values for polycrystalline and single crystal ice^30,31^. These two spectral components corresponding to a fast liquid-like and a slow ice-like dynamic sub-population of water molecules coexist down to the lowest temperature (193 K) explored in the present study, although the relative fraction of the Lorentzian component decreases rapidly with cooling. Similar observations were also made in previous studies of water under nanoconfinement in other porous materials such as in mesoporous silica MCM-41 and in proteins such as collagen, elastin and myoglobin^25,28^. Such ‘quasi’ two-phase spectra imply the presence of a rather broad distribution of rotational correlation times or activation barriers, where the spectral line shape is dominated by the slow and fast populations^32^. This type of dynamical behavior is unprecedented in bulk liquids, but it is the hallmark of glassy dynamics with strong static heterogeneity and a lack of significant diffusive exchange between the slow and fast sub-populations. As such, this static heterogeneity is consistent with a layered core-shell structure of water in nanopores.

**Figure 3:**
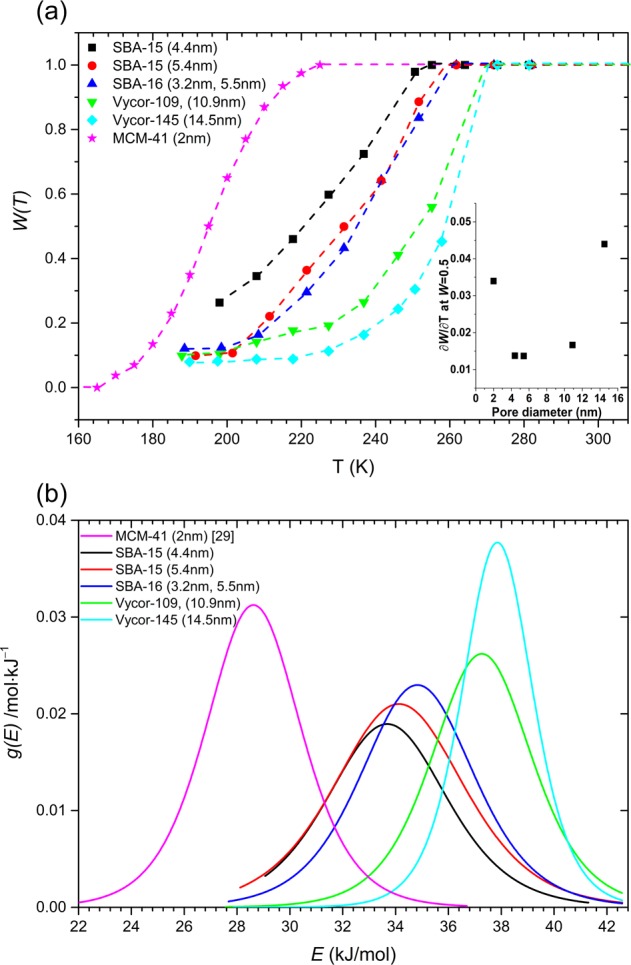
(**a**) Temperature dependence of the relative fraction of liquid-like dynamical population *W(T)* of D_2_O confined in different silica matrices. Inset shows variation of the slope $\partial W(T)/\partial T$ at *W* = 0.5, as a function of pore diameter. (**b**) Activation energy distribution *g(E)* for molecular dynamics of D_2_O confined in different silica matrices, obtained from the temperature derivative of *W(T)* curves in (**a**). For details of the calculation see text. *W(T)* data for MCM-41 is from^28^.

The relative fraction of the liquid-like dynamical population *W* can be obtained from the simulation of these ^2^H NMR spectra with a Lorentzian line and a Pake pattern (Fig. 2b). The temperature dependence of this fraction *W(T)* for all samples is shown in Fig. 3a and is compared with that reported in a previous study for D_2_O confined in 2.0 nm diameter pores in MCM-41^28^. The onset temperature where *W(T)* starts to decrease below 1, i.e. the solid-like sub-population starts to appear in the ^2^H NMR spectra, is consistent with the melting temperature from DSC measurement. With further reduction in temperature *W(T)* monotonically decreases following a sigmoidal kinetics until it reaches ~0.1 near 193 K for all samples. This limiting value of *W(T)* likely corresponds to the water molecules in the immediate vicinity of the pore wall that remain mobile down to 193 K. The most interesting result however, is the fact that the fast-to-slow sub-population conversion rate upon cooling is the highest for water confined in either the smallest (~2.0 nm) or the largest (≥10 nm) pores, while the rates are lower for confinement in intermediate-sized (4–6 nm) pores. This result is completely consistent with the core-shell model. Considering the pore diameters and the aforementioned thickness of the shell (~1 nm), one would expect that the vast majority of the water molecules reside in the shell for pores with diameter ≤3 nm, while they reside in the core for pores with diameter ≥10 nm. Therefore, these regions being characterized by a relatively narrow temperature range of melting/freezing, *W(T)* is expected to show a rapid drop with temperature on cooling. On the other hand, for intermediate pore diameters between 3 and 10 nm, the relative fractions of core and shell regions become comparable and the hypothesized gradual variation in the melting/freezing point across the core-shell boundary would lead to a gentle drop in *W(T)* on cooling. These expected variations in *W(T)* are indeed borne out in the experimental results as shown in Fig. 3a, thereby lending support to the “layer-by-layer” freezing model mentioned above.

Additionally, for weakly temperature-dependent distribution of energy barriers, following the Arrhenius law, it can be shown that the activation energy distribution *g(E)* is related to the derivative of *W(T)* with respect to temperature as^32^: $g(E)=\frac{\partial W(T)/\partial T}{\mathrm{ln}\left(\frac{{\tau }^{\ast }}{{\tau }_{0}}\right)}$. In this expression τ^*^is ~1/C_Q_, which is ~10^−6^s for ^2^H and *τ*_0_ is a phonon timescale (10^−13^ to 10^−14^ s), corresponding to an attempt rate. The temperature scale is converted to an energy scale using the Arrhenius relation $E=RT\,\mathrm{ln}\left(\frac{{\tau }^{\ast }}{{\tau }_{0}}\right)$ where *R* is the gas constant, τ^*^= 4.5*10^−6^s and *τ*_0_ = 10^−13^ s. As expected, the activation energy distribution becomes narrower for smaller and larger pores, compared to that for the intermediate pore sizes (Fig. 3b). However, the average activation energy ranges between ~28 and 38 kJ.mol^−1^, consistent with previous reports of activation energy measurements using NMR spin-lattice relaxation, dielectric relaxation and quasi-elastic neutron scattering techniques^13,27,33^.

## Core vs. shell dynamics and fragile-to-strong transition in confined water

**Figure 4:**
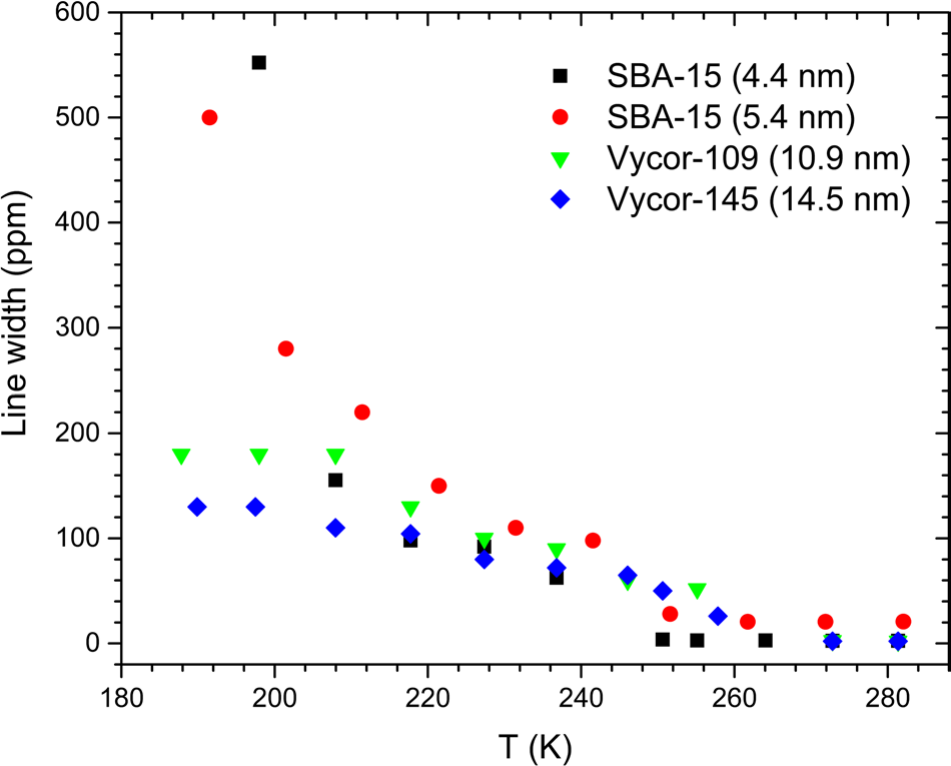
Full-width-at-half-maximum of the Lorentzian component in ^2^H NMR spectra of D_2_O confined in different silica matrices as a function of temperature.

The layer-by-layer freezing model also offers an explanation for the observation of an apparent transition in the temperature dependence of the relaxation time of water from a non-Arrhenius (i.e. fragile) to an Arrhenius (i.e. strong) behavior upon cooling^11,12^. As recently noted by Lederle *et al*^34^, with progressive freezing of nanoconfined water from the center towards the pore wall, the remaining liquid fraction becomes increasingly confined by the surrounding. This confinement is expected to be particularly severe in smaller pores. The width Δ of the Lorentzian ^2^H NMR line corresponding to the liquid-like dynamical population is a direct measure of the mobility of the water molecules in the liquid fraction. The temperature dependence of Δ for all samples is shown in Fig. 4, which increases with the lowering of temperature indicating that the mobility of the liquid fraction slows down with increasing solid-like fraction. This lowering in the mobility becomes rapid and strong as the pore size decreases (Fig. 4), implying increased effect of confinement on the remainder of the liquid fraction. As the length scale of this confinement becomes comparable to or smaller than that of the cooperatively rearranging regions responsible for structural or α-relaxation of the liquid, the effect of cooperative molecular motion would greatly diminish, and the dynamics would become Arrhenius^35^. Such a transition in the dynamical behavior upon strong nanoconfinement has been reported in the past for glass-forming liquids such as glycerol^36^. Therefore, we argue that the fragile-to-strong transition is a direct consequence of the layer-by-layer solidification behavior of water under strong nanoconfinement.

In summary we report a systematic study of the melting and freezing dynamics of D_2_O confined in the nanopores of solid silica matrices with pore sizes ranging between ~ 4–15 nm, using a combination of DSC and ^2^H NMR. In spite of the differences between the mesoporous silica and amorphous vycor in the nature of their internal pore surfaces and possibly in their interactions with water, the melting point depression in all materials obey a single Gibbs-Thomson relation. Moreover, for all confining media the results are generally consistent with a model of freezing characterized by strong spatially heterogeneous glassy dynamics. The extreme spatial heterogeneity in the dynamics is manifested in the very first observation of two distinct freezing points for water nanoconfined in interconnected pores of two different diameters in mesoporous silica SBA-16. The freezing likely starts at the center of the nanopores and proceeds gradually layer-by-layer, towards the periphery of the pore. This layer-by-layer freezing model that we propose also provides a credible explanation for an apparent fragile-to-strong transition in the temperature dependence of the relaxation time of nanoconfined water as reported in previous studies.

## Methods

### Sample preparation

Mesoporous SBA-15 and SBA-16 silica powders were synthesized according to acidic reaction methods as previously reported^37^. SBA-15 was synthesized by dissolving 4.0 g of triblock copolymer poly (ethylene oxide)20-poly(propylene oxide)70-poly(ethylene oxide)20 (Pluronic® P123, Aldrich), in 30 mL of water and 120 mL of 2 M HCl solution. Then, 9.1 mL of tetraethylorthosilicate (TEOS, Aldrich) was added to the solution, which was kept at 37 °C for 20 hours. Subsequently, the mixture was placed in an oven at 70 °C for hydrothermal treatment for 16 hours. This temperature was chosen to obtain SBA-15 samples with thick walls and to avoid formation of pores connecting the mesopore channels^38^. The resulting white precipitate was then washed with water and ethanol, dried in an oven at 70 °C and calcined at 500 °C for 3 hours. To synthesize SBA-16, 3.0 g of triblock copolymer poly (ethylene oxide)106-poly(propylene oxide)70-poly(ethylene oxide)106 (Pluronic® F127, Aldrich) were dissolved in 144 mL of water and 144 mL of 2 M HCl solution. Subsequently, 11 mL of butyl alcohol and 15 mL of TEOS were added. The solution was then kept in an oven at 45 °C for 24 hours, followed by a hydrothermal treatment 100 °C for 24 hours. The resulting white precipitate was then washed, dried, and calcined as described above for SBA-15.

The mesopore structure was determined by transmission electron microscopy (TEM) characterization. For TEM imaging, SBA-15 and SBA-16 powders were dispersed in ethanol. A small amount of this dispersion was placed on a TEM grid by drop-casting and dried in air. TEM images of mesoporous materials were taken by an FEI Tecnai 12 transmission electron microscope. Nitrogen adsorption/desorption isotherms were obtained using a Sorptometer (Gemini 5, Micromeritics) at 77 K. The mesoporous materials were degassed at 523 K for 6 hours at a pressure of 3 mTorr prior to adsorption. The specific surface areas of the samples were calculated by the Brunauer-Emmett-Teller (BET) method, and pore size distribution were calculated by the Density Functional Theory (DFT) method using Micromeritics software kernels. The average pore size of two different SBA-15 samples with hexagonally arranged cylindrical pores was found to be 4.4 and 5.4 nm, whereas in cage-structured SBA-16, large spherical pores (5.5 nm) are body-centered-cubic arranged and connected by narrow openings (3.2 nm). D_2_O was added to excess amount of as-prepared mesoporous silica powder and the mixture was stirred to obtain partially hydrated samples for DSC and NMR experiments.

The Vycor-109 and Vycor-145 porous glasses with average pore size of 10.9 nm and 14.5 nm, respectively, were obtained from Corning Inc. in the form of buttons of dimensions 6.9 mm diameter and 2.5 mm height by Corning Glass Works. These glasses were prepared from commercial Vycor (Corning code 7930) parent glass using extended heat treatments to coarsen the microstructure of the leachable phase followed by sequential etch treatments. Vycor-109 (145) was prepared by a heat treatment of the 7930 parent glass for 10 (80) h at 580 °C followed by cooling at 25(10) °C/h. These heat-treated samples were then immersed in 30 wt% NH_4_F solution for 5 minutes and subsequently directly transferred to 0.25 N HNO_3_ kept at 95 °C, and were etched for 2 h. Etched samples were rinsed at 95 °C in deionized water for 1 h and dried at 100 °C in an oven. The average pore size of these samples were determined using mercury porosimetry at 50% intrusion level. ‘Thirsty’ Vycor glass was obtained by baking all samples at 200 °C overnight to remove any absorbed organic impurities.

The thirsty Vycor glass buttons were immersed in pure D_2_O (Aldrich) for 10 minutes to obtain fully hydrated samples. These samples were subsequently dried at ambient condition for 1 h to remove any freezable bulk water on the sample surface through water evaporation, crushed into powder and used in DSC and NMR experiments.

### Differential scanning calorimetry

The DSC measurements were carried out using a Mettler Toledo DSC1 differential scanning calorimeter. About 10–25 mg partially hydrated samples were hermetically sealed in 40 μL aluminum pans. Scans were taken in a flowing nitrogen environment with a heating rate of 5 K/min from −100 °C to 25 °C. Melting point was determined to within ±2 °C as the onset of the melting endotherm. The degree of water loading was found to have negligible effect on melting temperature determination^14^. The temperature scale was calibrated to the onset of the melting endotherm of pure bulk D_2_O at 277.0 K.

### ^2^H Nuclear Magnetic Resonance spectroscopy

All ^2^H wideline NMR spectra of the hydrated samples were acquired using a Bruker Avance500 spectrometer equipped with a Bruker magnet operating at 11.7 T (^2^H Larmor frequency of 76.7 MHz). The powder samples were taken in 4 mm and 7 mm ZrO_2_ rotors and NMR spectra were collected using either a Bruker 4 mm triple-resonance probe or a Bruker 7 mm double-resonance probe. The temperature was controlled to within ±2 K using nitrogen gas boil-off from liquid nitrogen. A solid-echo pulse sequence (π/2−τ−π/2−acquisition) was employed for spectral acquisition with π/2 pulse length of 3.2 μs, τ = 10 μs and a recycle delay of 1 s. Each spectrum was obtained by Fourier transforming the average of 512 free induction decays. Multiple spectra, acquired with stepped offset in carrier frequency covering overlapping frequency ranges with uniform excitation, were stitched together to obtain the full spectral line shape at each temperature. All ^2^H NMR line shapes were simulated using the software DMFit^39^.

## Data Availability

All relevant data are available from the authors upon reasonable request.
